# NaCl induced salt adaptive changes and enhanced accumulation of 20-hydroxyecdysone in the *in vitro* shoot cultures of *Spinacia oleracea* (L.)

**DOI:** 10.1038/s41598-019-48737-6

**Published:** 2019-08-29

**Authors:** Niramaya S. Muchate, Nilima S. Rajurkar, Penna Suprasanna, Tukaram D. Nikam

**Affiliations:** 1 Department of Botany, Savitribai Phule Pune University, Pune, India; 2 Department of Environmental Science, Savitribai Phule Pune University, Pune, India; 3 Department of Chemistry, Savitribai Phule Pune University, Pune, India; 4 Nuclear Agriculture and Biotechnology Division, Bhabha Atomic Research Centre, Mumbai, India

**Keywords:** Plant physiology, Salt

## Abstract

Spinach (*Spinacia oleracea* L.) is a vegetable plant with high nutritional properties. In the present work, we studied responses of *in vitro* shoot cultures to salt stress (0 (control), 100, 200 and 300 mM NaCl) and salt stress-induced accumulation of 20-hydroxyecdysone (20E). Our results revealed that effect of low to moderate level of salinity stress (100–200 mM) was less pronounced on growth and tissue water content (TWC) of shoot cultures compared to higher salinity level (300 mM). The salt treated shoot cultures showed better osmotic adjustment in terms of significant accumulation of compatible solutes and total soluble sugars and also higher antioxidant enzyme activity. As the NaCl stress was increased, there was a corresponding linear raise in the Na^+^ accumulation while the contents of both K^+^ and Ca^2+^ decreased significantly. We also studied salt-stress induced accumulation of a bioactive compound; 20E and results showed that 200 mM salt treated shoot cultures accumulated significantly 2.9 fold higher 20E as compared to untreated shoot cultures. The results suggest that *Spinacia oleracea* exhibits considerable salt tolerance with better osmotic adjustment and can be considered a suitable candidate for the production of bioactive secondary metabolite.

## Introduction

Salinity stress exerts its negative effect on plant growth due the osmotic or ionic stress or nutritional imbalance^1^. Generally, plants adapt to saline condition by developing certain biochemical mechanisms such as synthesis of compatible solutes, alteration in activity of antioxidant enzyme and concentration of inorganic ions^2,3^. In addition, plants under saline conditions also produce secondary metabolites which have a role in defense against environmental stresses. Salt stress induced accumulation of polyphenols and flavonoids have been reported in *Cakile maritima*^4^ and *Hordeum vulgare*^5^. There is a need to screen for plant species that not only possess significant survival under salt stress with higher productivity but also synthesize certain bioactive metabolites. Such biopropsecting studies have gained considerable research focus in the recent years. In this regard, *Spinacia oleracea,* a herb of Chenopodiaceae, is chosen as it is an annual, rapid growing leafy vegetable. It is a rich source of minerals, vitamins, and edible proteins and 20-Hydroxyecdysone (20E), a secondary metabolite^6,7^. 20E plays an essential role in moulting, metamorphosis, embryonic and larval development of insects^8^ besides having therapeutic value in wound healing, performance-enhancing and anti-osteoporotic properties^9^. The 20E and its derivatives have also been reported effective in enhancing protein synthesis, improvement in human health and curing some of the disorders arising due to human immunodeficiency virus (HIV). It is also reported having antioxidant and tonic properties^10,11^.

In our previous work, we studied the response of *S. oleracea* to different level of salinity stress in pot experiment and showed that the *Spinacia oleracea* has good potential to desalinize the saline soil and also the salt stressed plant parts can be used for the production of 20E^7^. Despite such potential, plants grown *ex vitro* will have to be completely used in extraction of 20E demanding that sustainable systems are required for continuous production. In this regard, plant tissue culture technique is very useful for the production of bioactive metabolites as they are maintained under controlled environment for continued growth and biomass production besides for studying the salt-induced physiological and biological changes. The *in vitro* technique is more advantageous due to enhanced efficient and rapid isolation of secondary metabolites independent of ambient weather and microbial contamination^12^. It has been suggested that the plant tissue culture technique can assist the commercial scale production of secondary metabolites for a wide variety of pharmaceutical and industrial applications^13^. Although accumulation of bioactive 20E has been evidenced in different plant species, there is no report on the salinity induced 20E accumulation in *Spinacia oleracea in vitro* grown cultures. In the present work, we have studied the effect of different levels of salinity (NaCl) on the growth of *Spinacia oleracea* under *in vitro* controlled conditions to determine the extent of its salt adaptiveness based on osmolytes, antioxidant defense and Na^+^ sequestration. We have also shown salinity induced higher accumulation of a bioactive secondary metabolite 20E in shoot cultures.

## Material and Methods

### Establishment of *in vitro* shoots culture

Seeds of *Spinacia oleracea* were soaked (2 min) in sterilized distilled water containing 2–3 drops of Sovistin solution; followed by washing 3–5 times using sterilized distilled water. Then seeds were surface sterilized in 0.1% HgCl_2_ for 2 min and were washed with sterilized distilled water five times. All the operations were carried out in a sterilized condition on a laminar air flow table. The surface sterilized seeds were inoculated on Murashuge and Skoogs (MS) medium^14^ containing 0.3% sucrose and 0.8% agar. After seed germination, cotyledon was separated aseptically and transferred on MS medium containing sucrose (3%) and 2-isopentenyl Adenine (2ip, 20 µM) for shoot induction. The established explants of *Spinacea oleracea* were vertically cultured onto MS medium supplemented with 20 µM 2ip and 3% sucrose for shoot multiplication.

The medium pH was adjusted to 5.8 prior to addition of agar (0.8%) and autoclaving at 121 °C for 15 min. The shoot cultures were maintained throughout the experiment at 25 ± 2 °C temperature, 16-h photoperiod using cool white fluorescent light (40 µM m^−2^ S^−1^ irradiance) and 70% relative humidity. The incubated explants were sub-cultured regularly at an interval of 21 days for 6 months under control conditions to generate shoot cultures for further experiments.

### Salt (NaCl) stress treatment

For studies on *in vitro* NaCl stress tolerance, fresh, actively growing shoot cultures were transferred to the MS basal nutrient medium consisting of 20 µM 2ip supplemented with various concentrations of NaCl (0, 100, 200 and 300 mM). The nutrient medium pH was adjusted to 5.8 and solidified with 0.8% agar prior to autoclaving at 121 °C for 15 min. All the cultures were incubated at the controlled condition as described above. After 21 days of salt stress treatment, the growth, tissue water content, oxidative stress, compatible solutes accumulation, antioxidant enzymes and inorganic ion content were measured using standard methods as mentioned bellow.

### Biochemical assays

#### Growth analysis, leaf succulence and tissue water content

Growth attributes such as number of shoots and number of leaves per culture, fresh weight (FW) and dry weight (DW) were recorded. Explants from the salt stressed and control cultures were taken and weighed to get the FW. The biomass obtained from cultures were dired in an oven at 80 °C until getting the constant dry weight (DW). Following equations were used to determine the tissue water content (TWC %) and leaf succulence index. TWC % = [(FW-DW)/FW] *100 and, Ls (mg/cm^2^) = Leaf FW/leaf surface^15^.

#### Relative electrolytic leakage

The fresh tissue (1 gm) was cut into small pieces using surgical blade. The tissue pieces were suspended in distilled water (10 ml) and incubated for 24 h in the test tube at room temperature. After incubation, the initial electrical conductivity (EC_1_) was measured. Then, for ion release from the tissue, the samples were autoclaved at 121 °C temperature for 15 min. After cooling, the samples were used for measuring the final electrical conductivity (EC_2_). Following equation was used to determine the relative electrolytic leakage (REL) of the samples REL = (EC_1_/EC_2_) * 100^16^.

#### Lipid peroxidation assay

Lipid peroxidation activity in tissue sample was measured according to Heath and Packer method^17^ and presented in terms of Malondialdehyde (MDA) content. Fresh leaf samples were powdered in liquid nitrogen. The powder was homogenized in 5 ml of trichloroacetic acid (TCA). The homogenate was centrifuged at 10,000 rpm for 10 min at 4 °C temperature. From this 2 ml supernatant was taken and blended with 2 ml of 0.67% thiobarbituric (TBA). Then the mixture was heated at 100 °C for 30 min and the reaction was terminated by cooling the mixture on an ice bath. The mixture was centrifuge at 10,000 rpm for 2 min to remove suspended impurities. The absorbance of the mixture was measured at 532 nm. The mixture of 0.025% TBA in 10% TCA was used as blank. The malondialdehide content was calculated as µM g^−1^ FW using an extinction coefficient of 155 Mm^−1^ cm^−1^.

#### Proline

The quantitative estimation of free proline in shoot samples was carried out according to Bates *et al*. method^18^. In this method, 500 mg fresh tissue homogenized in 2 ml of 3%(w/v) of aqueous 5-sulphosalicyclic acid, then, homogenates were transferred to centrifuge tube and centrifuged at 10,000 rpm for 10 min at 4 °C temperature. Two milliliter of each supernatant transferred to test tube and mixed with 2 ml of glacial acetic acid and 2 ml of acid ninhydrin as a reagent. The test tube containing reaction mixture was heated at 100 °C in hot water bath for 1 h. The tubes were then cooled on ice bath to which 4 ml of toluene was added and the mixture was vortexed for 3 min at 30 rpm. After phase separation, upper layer was used for measuring the absorbance at 520 nm. A standard curve for proline was prepared using L-proline (Sgma-Aldrich) as standard.

#### Glycine betaine

The Grieve and Grattan method^19^ was used for Glycine betaine (GB) estimation. Fresh tissues (500 mg) were powdered in liquid nitrogen and then transferred to 20 ml of deionised water. The homogenate solution was placed on the rotary shaker at 80 rpm for 16 h at 25 °C temperature. Subsequently the filtered homogenates (500 µl) were diluted (1:1) with 2 N H_2_SO_4_ and the mixtures were cooled for 1 h in an ice water bath. Then 200 µl of I_2_-KI_2_ added to each sample and kept for 16 h at 4 °C temperature. The samples were centrifuged at 10,000 rpm for 15 min at 0 °C and periodide crystals were dissolved on addition of 1, 2- dichloroethane (9 ml). Mixture was placed in static condition for 2 h and absorbance of organic layer was measured at 365 nm. A standard curve developed with different concentration of glycine betaine.

#### Total soluble sugar

The quantitative estimation of total soluble sugar (TSS) content was carried out by anthrone method^20^. The fresh tissue samples (200 mg) were homogenized using ice-chilled ethanol (80%) and the homogenate was centrifuged at 5000 rpm for 10 min at 4 °C. Final volume of supernatant was made to 10 ml with ethanol (80%). The anthrone reagent (3 ml) was added to one ml of supernatant sample and the mixture was kept for 10 min at 100 °C in hot water bath. Then the mixture was transferred to an ice bath for termination of reaction. For quantification, the absobance was measured at 620 nm. The TSS content was determined from a standard curve prepared using D-glucose as standard.

#### Inorganic ion content

The oven dried tissue powder (100 mg) was diagested with 0.5% nitric acid and kept at 100 °C for 30 min. After digestion and decolorization the sample were used for estimation of Na^+^, K^+^ and Ca^2+^ content. The ion conent was measured by using atomic absorption spectrophotometer^21^.

#### Antioxidant enzyme activity

For preparation of the extracts, fresh leaf tissue (1 g) samples were homogenized using a chilled morter and pestle. Homogenisation was carried out on addition of 5 ml of ice-cold sodium phosphate buffer (50 mM, pH 7) containing EDTA (0.1 mM) and polyvinylpyrrolidone 1% (w/v). The homogenates were centrifuged at 15,000 rpm for 20 min at 4 °C and the supernatant was used as a source of crude enzymes. The content of enzyme protein was determined by following the Lowry method^22^. BSA was used as a source of standard protein.

Catalase (EC 1.11.1.6): The catalase (CAT) activity was measured according to Cakmak and Marschner^23^ with minor modifications. The reaction mixture (1 ml) consisting phosphate buffer (50 mM, pH 7), H_2_O_2_ (15 mM) and enzyme extract (50 µl) The decomposition of H_2_O_2_ and corresponding decrease in absorbance was recorded at 240 nm for 2 min at an interval of 15 s. The enzyme activity was calculated and presented as µKat at of CAT activity mg^−1^ protein (ɛ = 36 mM^−1^ cm^−1^).

Ascorbate peroxidase (EC1.11.1.11): The ascorbate peroxidase (APX) activity was assessed by following Nakano and Asada^24^. The 1 ml of reaction mixture prepared contains 50 mM phosphate buffer (pH 7), 0.5 mM ascorbate, 0.1 mMH_2_O_2_, 0.1 mM EDTA and 100 µl enzyme extract. The decrease in absorbance was measured at 290 nm at 15 s interval for 1 min. The activity was presented as µKat at of APX activity mg^−1^ protein (ɛ = 2.8 mM^−1^ cm^−1^).

Superoxide dismutase (EC 1.15.1.1): The Superoxide dismutase (SOD) activity was measured according to the method described by Becana *et al*.^25^. The reaction medium (3 ml) was prepared on addition of 50  mM phosphate buffer (pH 7.8), 2.2 µM riboflavin, 14.3 mM methionine, 0.1 mM EDTA and 50 µl enzyme extract. The reaction medium for all samples including light blank (without enzyme extract) were placed at a distance of 30 cm from the light source and exposed to light for 30 min. At the same time, blank sample (the reaction mixture + 100 µl enzyme extract) was kept in the dark for 30 min. The reduction in NBT was determined by recording the change in absorbance at 560 nm and expressed U SOD per mg protein per minute (1 U SOD is equivalent to an amount that produces 50% inhibition of NBT) and expressed per mg protein per minute.

Guaiacol peroxidase (EC 1.11.1.7): The guaiacol peroxidase (GPX) activity was examined following the method of Hemeda and Klein^26^. The reaction medium (1 ml) contained 50 mM phosphate buffer (pH 6.6), 1% guaiacol (w/v), 0.3% H_2_O_2_ and 100 µl enzyme extract. Change in absorbance was measured at 470 nm and activity expressed in units per mg protein per min. (One µmole of guicol oxidized per min = one unit).

Glutathione reductase (EC 1.6.4.2): The activity of glutathione reductase (GR) was measured in a reaction mixture that consisted of 50 mM phosphate buffer (pH 7.5), 1 mM EDTA, 3 mM DTNB in 0.01 mM phosphate buffer (pH 7.5), 0.1 mM H_2_O_2_, 2 mM NADPH, enzyme extract and GSSG. The change in absorbance was recorded for 2 min at 421 nm and expressed per mg protein per min (1 µmole of GSSG reduced per min = 1 unit)^27^.

#### 20-Hydroxyecdysone analysis

Control and treated shoots were used for 20E estimation. HPTLC was performed using Muchate *et al*. method^28^. The shoot culture samples were powdered in liquid nitrogen. Dry powder (1 g) of each sample was suspended in ethanol (3 ml) and kept at room temperature for 12 hr. Then, the samples were centrifuge at 10,000 rpm (revolution per minute) and supernatant was filtered through 0.22 µM filter. The filtrate was used for analysis. For preparation of standard stock solution, 2.15 mg of standard 20E (purity93%) (Sigma-Aldrich) was dissolved in 2 ml of ethanol (99.9%). The 50 µl of stock solution was diluted by addition of 950 µl of ethanol and used as working standard solution (20E concentration 1 µl = 50 ng) for analysis of 20E using HPTLC.

The HPTLC plate was rinsed with ethanol-ethyl acetate-water (8: 2: 0.5, v/v/v) and then, spots were loaded with various concentrations of standard (50,100, 200, 300, 400, and 500 ng per spot) and experimental samples of 20E. For development of plate, the mixture of chloroform-methanol-benzene (12.5: 2.5: 1.5, v/v/v) was used as a solvent system. The dried developed plate was analyzed for the content of 20E in each spot using TLC scanner based on fluorescent quenching at 254 nm.

#### Statistical analysis

The experiments were carried out using completely randomized design (CRD) with at least three replicates. The one-way analysis of variance (ANOVA) done using SPSS 20.0. The mean comparisons were carried out using Duncan’s multiple rang test (P ≤ 0.05).

## Results

### Growth and water status

**Figure 1:**
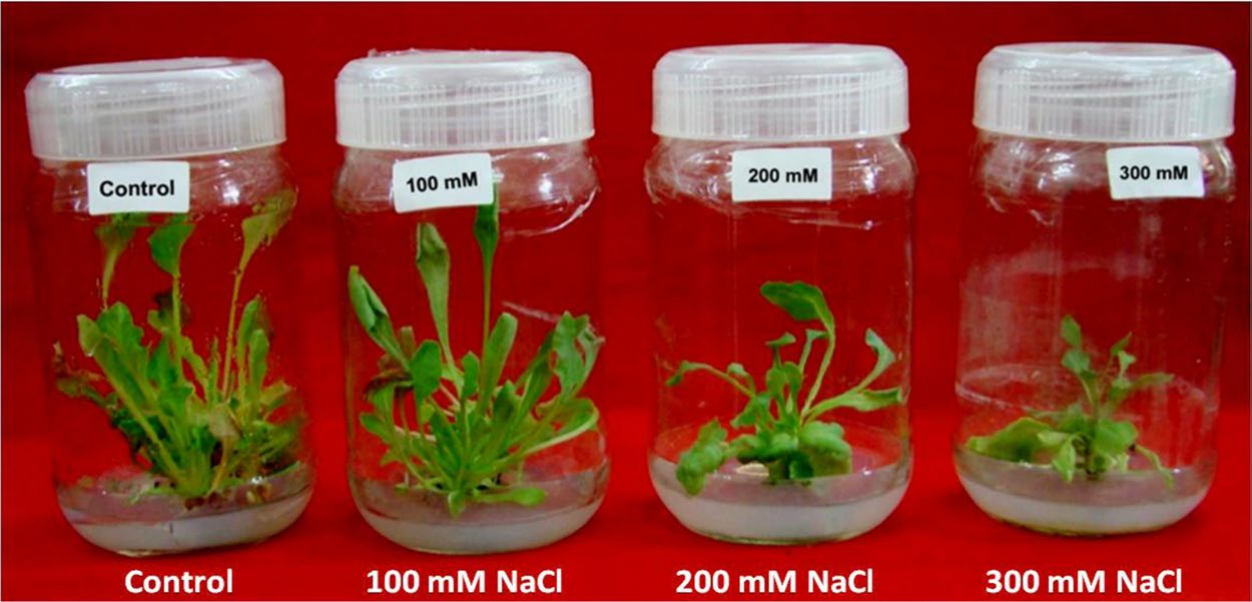
Effect of NaCl on growth of *in vitro* shoot cultures of *Spinacia oleracea*. The *in vitro* shoots were cultured onto the MS medium supplemented with 20 µM 2ip [6-(γ,γ-Dimethylallylamino) purine] and different concentrations of sodium chloride (NaCl) (0, 100, 200 and 300 mM).

Shoot cultures subjected to treatments of salt (NaCl) exhibited decline in shoot number, leaves per culture, FW and TWC (Table 1, Fig. 1). Significant decline in number of shoots and number of leaves per culture was recorded with increase in NaCl concentration. The number of shoots (6.0 ± 0.0) and number of leaves (29.0 ± 0.6) were higher in control cultures after 21 days of incubation whereas salt treated cultures showed a gradual decrease in FW, FW/DW and TWC per shoot culture with increase in salinity however, shoot survival was noted up to 300 mM NaCl.

**Table 1:** Influence of NaCl stress on growth of *in vitro* regenerated shoots of *Spinacia oleracea*.

NaCl (mM)	No of shoots/explant	No of leaves/shoot	FW (g) of shoots/culture	DW (g) of shoots/culture	FW/DW ratio of shoots/culture	TWC (%) of shoots/culture
0	6 ± 0.0a	29.0 ± 0.6a	2.72 ± 0.19a	0.13 ± 0.01b	21 ± 1a	95.2 ± 0.2a
100	4 ± 0.1b	23.3 ± 0.9b	1.84 ± 0.14b	0.25 ± 0.02a	7.4 ± 0.1b	86.5 ± 0.2b
200	3 ± 0.1c	19.3 ± 0.3c	1.35 ± 0.13c	0.22 ± 0.02a	6.1 ± 0.5bc	83.5 ± 1.3b
300	1 ± 0.0d	14.0 ± 0.6d	1.06 ± 0.05c	0.23 ± 0.01a	4.7 ± 0.4c	78.5 ± 1.8c

Data represent the means of three replicates with standard error. Values sharing the common letters are not statistically different at P < 0.05.

### Oxidative damage

**Figure 2:**
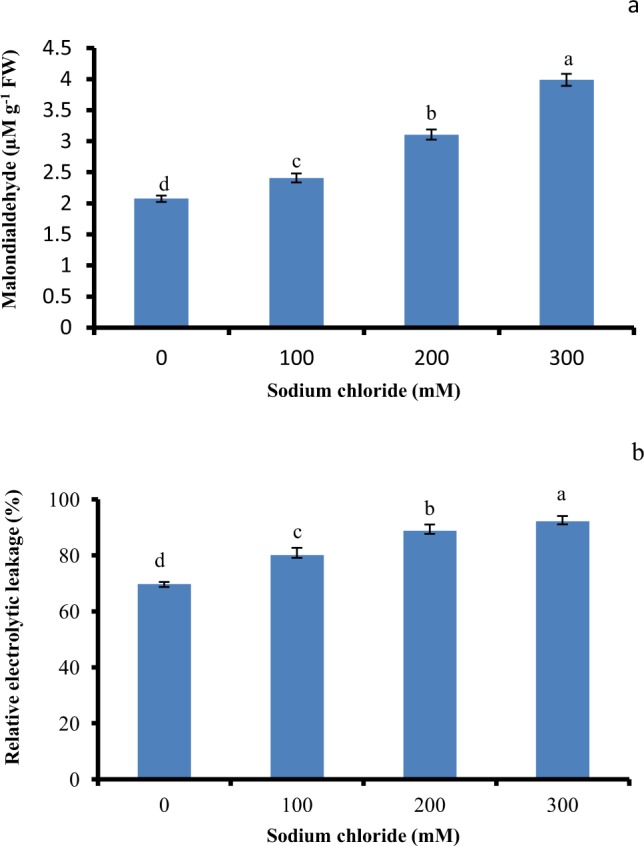
Effect of salinity stress on oxidative damage in *in vitro* shoot cultures of *Spinacia oleracea*. (**a**) Lipid peroxidation in terms of Malondialdehyde (MDA) content, (**b**) relative electrolyte leakage (REL). The vertical error bars with different letters represent the significant differences between treatment at p = 0.05 using DMRT.

Salt-induced oxidative damage in the salt treated shoot cultures was determined by measuring the MDA content and REL (Fig. 2). Compared to control, the MDA content of the shoots grown at 100 mM NaCl were not affected by salt stress (Fig. 2a). However, further increase in salt levels resulted in significantly enhanced MDA content. The shoots subjected to the treatment with 100 and 200 mM NaCl showed a slight increase in REL over the control (Fig. 2b) whereas MDA content and REL (%) were 1.8 and 1.3 folds in shoots treated with 300 mM salt stress respectively.

### Osmotic adjustment

**Figure 3:**
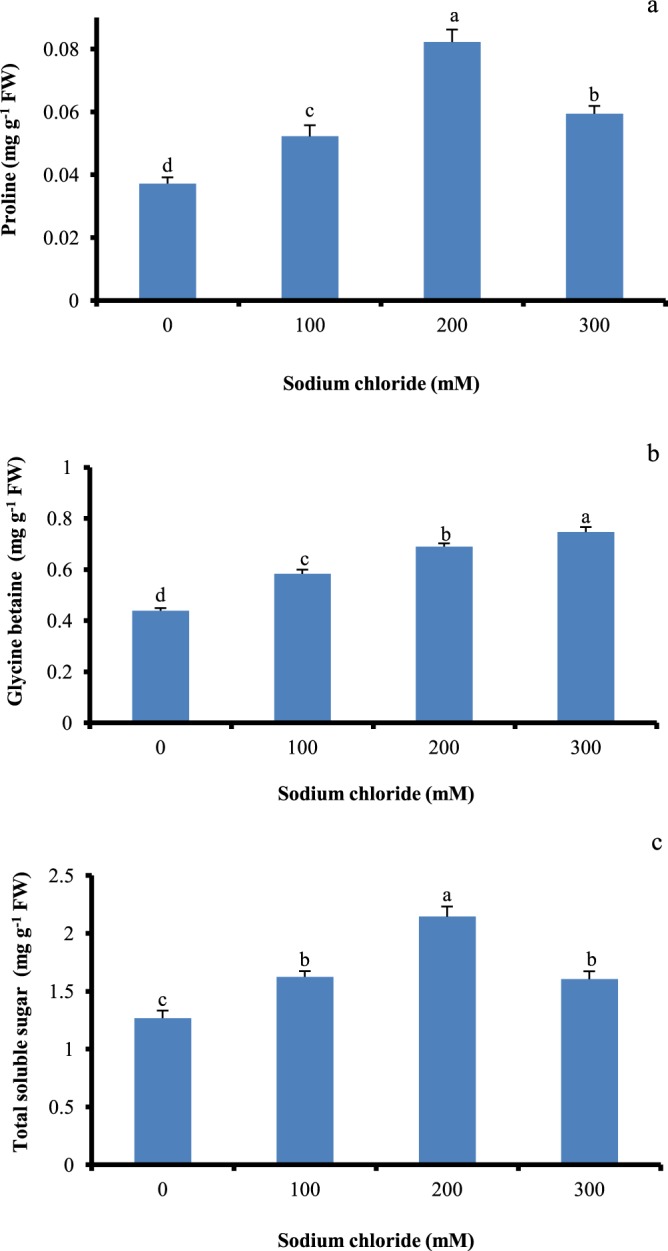
Effect of salinity stress on osmolytes accumulation in *in vitro* shoot cultures of *Spinacia oleracea*. (**a**) proline, (**b**) glycine betaine (GB), and (**c**) total soluble sugars (TSS). The vertical error bars with different letters represent the significant differences between treatment at p = 0.05 using DMRT.

Significant proline accumulation was noted in salt treated shoot cultures and it was considerably high at 200 mM salt stress (Fig. 3a). Glycine betaine accumulation was noted low in control, whereas marked increased was recorded in salt treated (100–300 mM) shoot cultures (Fig. 3b). Over the control, significant increase in TSS content was observed in all the salt treated shoots (Fig. 3c). Shoots treated at 200 mM salt stress exhibited 1.6 fold higher TSS in comparison with non-treated control cultures.

### Antioxidant enzyme activity

In the present study, compared to control, the salt treated shoots showed enhanced activity of antioxidant enzymes (Table 2). The activity of SOD was enhanced with an increase in salt stress and it was highest in shoot cultures treated with 300 mM salt. Activity of CAT also showed a similar trend and it was enhanced significantly in the shoot cultures grown from 100–300 mM salt. No significant change in activity of GPX was observed in shoots treated with 100 mM NaCl while activity of GPX increased significantly in shoot cultures subjected to200 and 300 mM NaCl in comparison to the control (Table 2). Control shoots cultures showed less APX activity in contrast to treated samples and it was significantly higher in cultures grown at 100–300 mM salt. The gradual increase in GR activity was observed in shoot cultures exposed to increased levels of salt (200 mM).

**Table 2:** Effect of salinity stress on antioxidant enzyme activity in *in vitro* regenerated shoots of *Spinacia oleracea*.

NaCl (mM)	SOD (U/mg protein)	CAT (µKat mg^−1^ protein)	GPX (µKat mg^−1^ protein)	APX (µKat mg^−1^ protein)	GR (µKat mg^−1^ protein)
0	11.3 ± 1.2d	0.28 ± 0.05c	0.38 ± 0.02c	0.053 ± 0.002c	0.04 ± 0.004b
100	17.4 ± 1.0c	0.39 ± 0.03bc	0.48 ± 0.05c	0.083 ± 0.003a	0.07 ± 0.008a
200	21.9 ± 1.6b	0.46 ± 0.03b	0.65 ± 0.05b	0.088 ± 0a	0.08 ± 0.007a
300	26.9 ± 1.1a	0.54 ± 0.06a	1.13 ± 0.01a	0.065 ± 0.0b	0.05 ± 0.002b

Values are mean of three replicates with standard error. Values sharing the common letters are not statistically different at P < 0.05.

### Ion content

**Figure 4:**
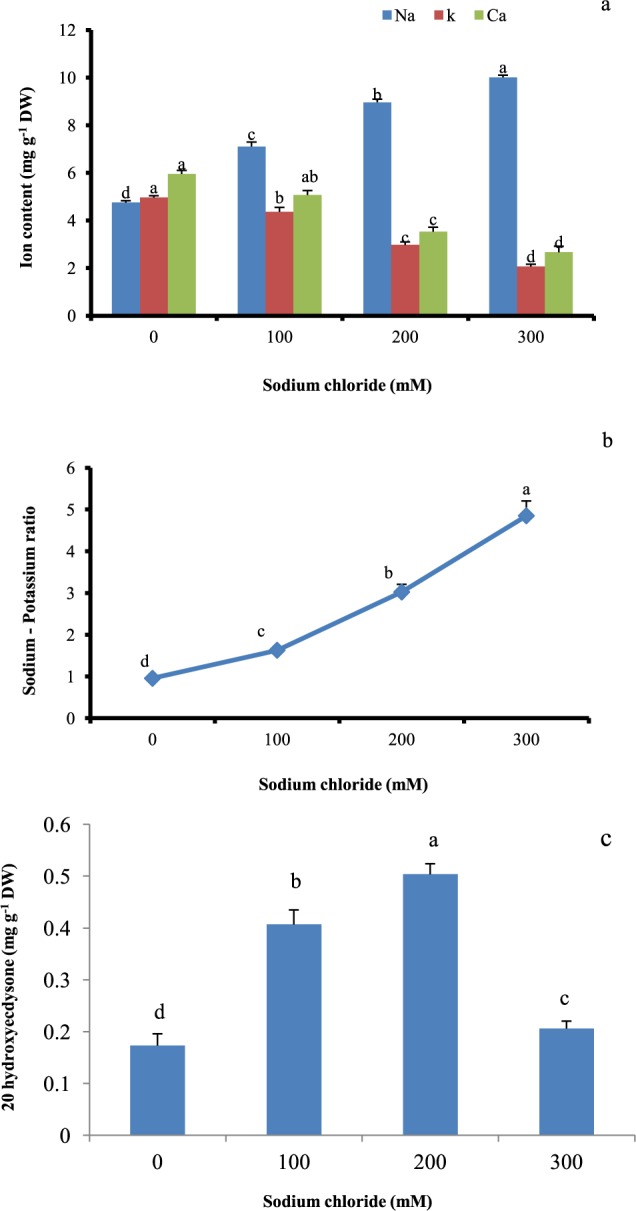
Effect of salinity stress on (**a**) mineral ion content, (**b**) ratio of sodium to potassium and (**c**) accumulation of 20-hydroxyecdysone (20E) in *in vitro* shoot cultures of *Spinacia oleracea*. For mineral ion content the statistical analysis was done per ion. The vertical error bars with different letters represent the significant differences between treatment at p = 0.05 using DMRT.

Sodium ion levels in stressed shoot cultures were significantly higher with increase in salt level than the control (Fig. 4a). Higher Na^+^ accumulation (1.9 fold) was observed in shoot cultures grown at 300 mM NaCl (Fig. 4a). On other hand, potassium and calcium ion concentrations were found to decline with increase in levels of NaCl (Fig. 4a). Over the control, all the treated shoot cultures showed considerable Na/K and significantly higher Na/K was observed in plants grown at 300 mM of salt (Fig. 4b).

### 20-hydroxyecdysone accumulation

The results on the influence of different levels of NaCl on 20-hydroxyecdysone accumulation in shoot cultures are shown in Fig. 4c. The 20E content in the salt treated shoots was significantly higher than in non treated control shoots (Fig. 4c). The maximum content of 20E was observed in shoots subjected to the treatment of 200 mM salt as compared to low content of 20 E (0.20 mg/g DW) under treatment with 300 mM NaCl.

## Discussion

Salinity through osmotic and ionic stresses exerts negative effect on growth, development and plant metabolic machinery^1^. Spinach is mild salt tolerant and has been demonstrated that the salt accumulation take place in leaf cells and not in apoplast^29^. Although response of Spinach to salt stress at whole plant level has been shown to involve osmotic adjustment through glycine betaine accumulation^30^ however, less is known about the involvement of osmolytes, antioxidant enzyme and inorganic ion at cellular level. In our study, decrease in the number of shoots and leaves, FW, FW/DW and TWC (%) was observed in salt treated shoot cultures as compared to the control. Spinach is a leafy vegetable and when grown in normal field conditions has a water composition in the range of 91 to 93%. Since closed culture vessels are used for *in vitro* cultures, the humidity is often very high 100% surrounding the shoot cultures. This could be one of the reasons for high water content in the *in vitro* raised shoots. In the present investigation, the experiments were performed in close culture vessel and under *in vitro* conditions. Therefore, this might be the reason for high water accumulation in the control aerial parts of the Spinach plants. At low to moderate level of salinity stress (100–200 mM) growth of shoot cultures were less affected as compared with higher salinity levels (300 mM), however, shoots showed continued growth. In our previous work, response of pot-grown mature plants of *S. oleracea* exposed to 4–12 dS/m of soil EC showed slight reduction in growth at low level of salinity (4 dS/m EC) whereas but growth was significantly influenced at 12 dS/m^7^. Decline in growth and TWC (%) in the salt treated shoot cultures might be associated with the initial osmotic stress followed by ionic stress. Increased osmotic potential of saline medium affects The water and nutrient uptake of the plants was generally influenced by the increased osmotic potential of the saline medium. This might results in reduced metabolic activity necessary for the growth^31^. Bracci *et al*. reported shoot growth inhibition of *Olea europaea* cultivars upon exposure to salinity stress^32^. A similar trend in reduction in shoot number under salt stressed condition is reported in mulberry^31^. However, in the present study, linear increase in DW was noted with increasing salinity stress which could be due to the Na^+^ accrual. The accumulation of Na^+^ ions and increased leaf thickness in Spinach may be associated with increase in cell size instead of cell number^33^.

Salinity stress promotes the formation of ROS species which causes oxidative damage in the chloroplast^34^. The MDA content and REL, an output of the oxidative stress and membrane damage were used to measure the intensity of membrane damage in shoots exposed to salinity stress. Increased NaCl concentrations augment the MDA content and REL (%) than control shoots. Statistically, significant MDA and REL (%) under different levels of salinity might be linked with the damage to the membrane. The differential trend was also observed in Spinach plant grown at elevated levels of soil salinity (0.4, 4, 8 and 12 dS/m). Increases in MDA content and REL (%) were not observed at low EC 4 dS/ m whereas increased levels were recorded at higher EC (8 and 12 dS/m) than the control suggesting higher membrane damage and lipid peroxidation^7^. This type of response might be associated with the increased H_2_O_2_ which could be drastically enhanced under salinity stress among the plant species that undergo higher lipid peroxidation^35^.

Plants synthesize osmolytes like proline, glycine betaine and certain proteins which minimize the influence of ROS species formed due to salinity stress^36^. In our study, compared to untreated control, statistically significant elevation in proline, TSS and glycine betaine accumulation was observed in salt treated shoot cultures. Additionally, higher content of proline and TSS was observed in shoots cultures exposed to 200 mM salt stress which might be associated with the more energy utilization towards the osmolytes synthesis and this could result in decreased growth. Similarly, significant induction of glycine betaine was seen in *Spinacia oleracea* plants treated with salt stress^30^. Muchate *et al*.^7^ has previously reported the accumulation of 3–8 fold higher proline in mature potted plants grown at EC 8 dS/m, compared to the control. In addition to this, significant increase in content of TSS and an osmolyte glycine betaine was recorded in salt treated plants. It is interesting to note that, increase in sugars content in plants under salinity stress may be linked with maintenance of osmotic and water balance in the cells^36^. The large increase in content of glycine betaine and proline was observed in shoots of *Citrus macrophylla* exposed to salinity stress^37^. The higher accumulation of glycine betaine in plants is correlated to the enhanced tolerance to the stress^38^. Formation of reactive oxygen species (ROS) in response to the salinity stress causes the oxidative damage in plant cell. At the same time detoxification of ROS is carried out by increase in activity of SOD, APX, CAT, GR, and GPX. SOD activity is noted to be the primary line of defense system against ROS^34^. In the present study, the SOD and CAT activity enhanced with an increasing NaCl concentration under stress treatment, compared to the control and it was highest in shoot cultures treated with 300 mM salinity. SOD activity was considerably increased in *Bruguiera gymnorrhiza* and *Bruguiera parviflora* by the response to salt stress^39^. The H_2_O_2_ produced during oxidative reaction by SOD under salt stress is further used by enzymes such as CAT, APX and GPX^40^. The functioning of CAT and GPX enzyme together with SOD plays a key essential role in ROS scavenging process^41^. From the ROS scavenging enzymatic machinery, catalase plays a significant role in regulating the H_2_O_2_ level. In the current study, an increase of CAT activity by 1.9 fold, was observed at 300 mM salt, over the control. Similarly, in rice seedlings, increased CAT activity at 300 mM salt stress was observed^42^. Increased catalase activity contributes to maintain the cellular H_2_O_2_ level^43^. Improved APX activity is a good evidence of salinity stress tolerance in plants. APX is the most important reducing substrate that carries the dismutase of H_2_O_2_ to water^43^. Our data showed the significant APX activity in shoots cultured on 100–300 mM NaCl, over the control. Exposer to salinity stress, promote the APX activity in different plants such as tomato^41^, wild beet^44^, and sesame^45^. In the current study, the existence of NaCl in the growth media considerably stimulated the activity of GPX and GR enzyme in Spinach shoots in comparison to the control. GPX participates in numerous physiological processes and characterized as an electron donor^46^. In our previous work, response of *S. oleracea* to various levels of soil salinity (0.4, 4, 8 and 12 dS/m) also showed higher antioxidant enzyme activity compared with the control plants^7^. Results from this study revealed that salinity stress induces the activity of SOD, CAT, APX, GPX and GR in *in vitro* shoot cultures indicating an efficient antioxidative defense mechanism.

Ion-specific responses impose toxic effects on growth development. The concentration of sodium and potassium ions is crucial for the differential growth of the shoot in the saline environment. In the current study, Na^+^ ion content and Na^+^/K^+^ content in the shoot cultures were progressively enhanced with increasing salt stress as compared with the control however K^+^ and Ca^+2^ content shows the exactly negative trend. Similarly, increased Na^+^ content was noted in a number of plants such as rice^47^ and *Sesuvium*^48^. There might be correlation existing between sequestration of Na^+^ ion and osmotic adjustment. In the presence of salinity stress, imbalance exist between Na^+^ uptake and K^+^ homeostasis and also this affects the ratio of K^+^/Na^+^. In a number of plants, ion antagonism was observed between Na^+^ and Cl^-^ ions and uptake of Ca^2+^, K^+^, and Mg^2+^ ions. Decline in K^+^ concentration and high ratio of K^+^/Na^+^ in many of the species under salt stress has been shown as the tolerant response^34,49^.

Biotic and abiotic environmental factors are responsible for stimulation of biosynthesis and accumulation of bioactive metabolites in plants. On exposure to salinity stress, plant growth is affected due to ion toxicity but stimulative effect of salinity on production of bioactive and other metabolites has been reported in some plants^50^. In the current study, *S.oleracea* shoots were exposed to the different levels of salinity stress and observed for accumulation of 20-hydroxyecdysone (20E). 20E is known to influence embryogenesis, moulting, metamorphosis, embryonic and larval development of insects^8^. In the current study, higher accumulation of 20E was observed in shoot cultures exposed to salinity stress and it was significantly higher in shoots cultured on 200 mM salt. At higher salinity levels (300 mM) the 20E accrual was less as compared with other salt concentration (100–200 mM) however it was significant in comparison to the non treated control cultures. The higher synthesis 20-hydroxyecdysone in Spinach shoot cultures might be linked with the prevention of membrane damage induced by salinity stress. The different stress factors affect the biosynthesis of secondary and other metabolites like flavonoids as they play a significant role in tolerance and adaptation of plants to overcoming adverse stress condition. In soybean callus cultures, salinity stress improved the content of alkaloids, saponin, flavonoids, flavones and flavonols and many other secondary metabolites^51^. Similarly, salt stress enhanced the accumulation of hyoscyamine and scopolamine in root cultures of *Datura metal* L^52^. Our results show that the *in vitro* salt treated shoot cultures of Spinach have the potential for the production 20E, a commercially important secondary metabolite. The development and use of *in vitro* culture system for the production of bioactive and other metabolites can be advantageous as production and scale-up can be controlled under *in vitro* conditions, independently from climate and soil factors. Considering the need for alternate solutions for sustainable agriculture, plants like *Spinacia oleracea* can be potentially useful for different purposes of phytodesalination and bioprospecting for industrial use^7^.

## Conclusion

In conclusion, *Spinacia oleracea* showed high level of salt tolerance with survival up to 300 mM salt. The synthesis of compatible solutes such as proline, glycine betaine and TSS was observed in the shoot cultures as the main adaptive mechanism contributing to salt tolerance. In addition, the higher activity of antioxidant machinery CAT, GR, SOD, APX and GPX was observed in salt treated shoot cultures suggesting their role in stress tolerance. Increased Na^+^ sequestration and Na^+^/K^+^ ratio was evident with increasing salinity stress in the shoot cultures. The shoot cultures also showed significant accumulation of 20E, an insect moulting hormone suggesting that *Spinacia oleracea* can be useful for the production of 20-hydroxyecdysone. Overall, the study highlights use of *Spinacia oleracea* as the candidate plant for biosaline agriculture with commercial benefits.
